# Oxidative Stress and MicroRNAs in Endothelial Cells under Metabolic Disorders

**DOI:** 10.3390/cells12091341

**Published:** 2023-05-08

**Authors:** Morgan Minjares, Wendy Wu, Jie-Mei Wang

**Affiliations:** 1 Department of Pharmaceutical Sciences, Eugene Applebaum College of Pharmacy and Health Sciences, Wayne State University, Detroit, MI 48201, USA; morgan.minjares@wayne.edu; 2 Vera P Shiffman Medical Library, Wayne State University, 320 E Canfield St., Detroit, MI 48201, USA; wendy.wu@wayne.edu; 3 Center for Molecular Medicine and Genetics, Wayne State University, 320 E Canfield St., Detroit, MI 48201, USA; 4 Barbara Ann Karmanos Cancer Institute, 4100 John R St., Detroit, MI 48201, USA

**Keywords:** endothelial cells, oxidative stress, ROS, cardiovascular, metabolic disorders, microRNAs

## Abstract

Reactive oxygen species (ROS) are radical oxygen intermediates that serve as important second messengers in signal transduction. However, when the accumulation of these molecules exceeds the buffering capacity of antioxidant enzymes, oxidative stress and endothelial cell (EC) dysfunction occur. EC dysfunction shifts the vascular system into a pro-coagulative, proinflammatory state, thereby increasing the risk of developing cardiovascular (CV) diseases and metabolic disorders. Studies have turned to the investigation of microRNA treatment for CV risk factors, as these post-transcription regulators are known to co-regulate ROS. In this review, we will discuss ROS pathways and generation, normal endothelial cell physiology and ROS-induced dysfunction, and the current knowledge of common metabolic disorders and their connection to oxidative stress. Therapeutic strategies based on microRNAs in response to oxidative stress and microRNA’s regulatory roles in controlling ROS will also be explored. It is important to gain an in-depth comprehension of the mechanisms generating ROS and how manipulating these enzymatic byproducts can protect endothelial cell function from oxidative stress and prevent the development of vascular disorders.

## 1. Introduction

Reactive oxygen species (ROS) are byproducts of enzymatic reactions, composed of both free radical and non-free radical oxygen intermediates, and are generated in numerous cell compartments such as the endoplasmic reticulum (ER), mitochondria, and cell membrane [1,2]. At physiological concentrations, ROS are generated by several sources, including uncoupled endothelial nitric oxide synthases (eNOS), NADPH oxidases (NOX), xanthine oxidases (XO), cyclooxygenases, and the mitochondrial electron transport chain [3]. Antioxidant enzymes such as superoxide dismutase (SOD), catalase (CAT), and peroxidase (POD) are required to manage the production of ROS for cellular health [4,5]. 

Cardiovascular (CV) diseases, the leading causes of death worldwide, act to remodel blood vessels and restrict blood flow to the heart and nervous system [6]. Oxidative stress contributes to the development of CV risks and disorders, including hypertension, atherosclerosis, diabetes mellitus (DM), cardiomyopathy, obesity, and congestive heart failure [7,8]. The endothelium not only regulates the passage of nutrients between tissues, forms the inner lining of all blood vessels, and manages the protective barrier properties of the vascular system [9,10], but also modulates vascular growth and permeability, tissue metabolism, immune responses to inflammatory stimulation, stem cell recruitment, and regulation of vascular tone [11,12]. Endothelial cells (ECs) also secrete a wide range of cytokines and growth factors that regulate various physiological activities in autocrine, paracrine, and endocrine manners. One of the important molecules that ECs produce is nitric oxide (NO), generated by endothelial NO synthase (eNOS). NO is a vasoactive molecule that participates in vascular remodeling, vasodilation, platelet aggregation and adhesion, clot formation, and renal hemodynamics [13]. ROS serve as important second messengers for signal transduction and aid in modulating EC activation, proliferation, and angiogenesis [14,15]. However, under CV risks or disorders when the accumulation of ROS exceeds the buffering capacity of antioxidant enzymes, oxidative stress and molecular damage occur, leading to early apoptotic death and genomic alteration in ECs [16]. ECs then switch from a vasodilative, anti-inflammatory, anticoagulant environment to a vasoconstrictive, proinflammatory, and procoagulant environment, with decreased bioavailability of nitric oxide (NO) and overproduction of superoxide (O_2_^−^), leading to impaired cellular repair [17,18] and endothelial dysfunction. 

Extensive studies have investigated the therapeutic potential of various antioxidant therapies in ECs [6], with recent studies turning to the investigation of microRNAs (miRNA). MicroRNAs are short, non-coding RNA molecules that silence post-transcriptional target genes by promoting messenger RNA (mRNA) cleavage and/or inhibition of protein translation [19,20]. Emerging evidence has suggested that ROS and microRNAs co-regulate each other. ROS dysregulate the expression of miRNAs in vascular and immune cells, while miRNA manages oxidative stress by targeting specific mRNAs [21]. Due to the biodistribution of these molecules and the presence of multiple mRNA targets for a single microRNA, mechanistic approaches for reducing excessive oxidative stress without impacting other pathways have been evolving [22]. This review will discuss the aspects described above, including a detailed evaluation of ROS and CV disorders where EC dysfunction plays a pivotal role, metabolic syndromes induced by ROS, and future perspectives for microRNA treatments and prevention of drug-induced oxidative stress.

## 2. Generation of ROS in Endothelial Cells

**Figure 1:**
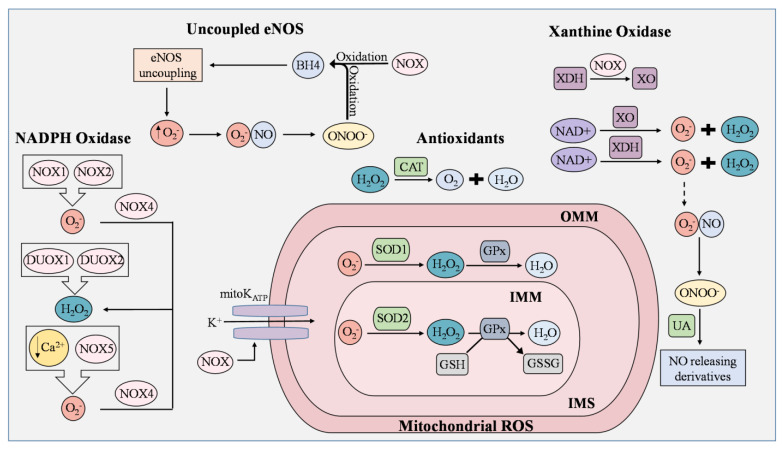
ROS generation and removal in endothelial cells. Reactive oxygen species (ROS) are mainly derived from uncoupled endothelial nitric oxide synthase (eNOS), NADPH oxidase (NOX), mitochondria, and xanthine oxidase (XO). Antioxidants that regulate ROS include catalase (CAT), superoxide dismutase 1 (SOD1), superoxide dismutase 2 (SOD2), and uric acid (UA). During eNOS uncoupling, superoxide (O_2_^−^) is produced in abundance rather than nitric oxide (NO). Superoxide then reacts with NO to generate peroxynitrite (ONOO^−^), which rapidly oxidizes tetrahydrobiopterin (BH4), thereby maintaining the state of eNOS uncoupling. ROS produced from NOX isoforms are involved in proliferation, migration, and differentiation in ECs, and are known to cause mitochondrial DNA damage, induce oxidative inactivation of BH4, stimulate the conversion of xanthine dehydrogenase (XDH) to XO, and manipulate the opening of the mitochondrial ATP-sensitive K+ ion channel (mitoKATP). NOX1 and NOX2 generate superoxide as opposed to NOX4, which converts superoxide to H_2_O_2_. In low concentrations of Ca^2+^, NOX5 enhances ROS production in ECs. Dual oxidase 1 (DUOX1) and dual oxidase 2 (DUOX2) possess roles in immune response and cell differentiation and are responsible for H_2_O_2_ generation. Leakage of electrons from the mitochondrial electron transport chain generates superoxide, but the presence of SOD1 and SOD2 prevents the buildup of damaging mitochondrial ROS (mROS). In the inner mitochondrial matrix (IMM), superoxide is converted to H_2_O_2_ by SOD2, while superoxide in the intermembrane space (IMS), which is between the IMM and outer mitochondrial matrix (OMM), is converted by SOD1. H_2_O_2_ is reduced to H_2_O by oxidizing glutathione (GSH) into glutathione disulfide (GSSG). This reaction is catalyzed by glutathione peroxidase (GPx). Catalase is an Fe-containing enzyme that catalyzes hydrogen peroxide into H_2_O and molecular oxygen. XO and XDH are the two interconvertible forms of xanthine oxidoreductase (XOR) where NAD+ is used to generate superoxide and peroxide. Under normal physiological conditions, UA possesses antioxidant activity and can aid in the regulation of ROS generated by XOR by reacting with ONOO^−^ to form NO-releasing derivatives.

ECs actively respond to hemodynamic changes and blood-borne signals and facilitate the exchange of oxygen, nutrients, solutes, hormones, and macromolecules between the blood and surrounding tissues, making them essential in the management of metabolic homeostasis [23]. Production of ROS by ECs is a required process for normal cellular function and can be triggered by oxidants produced by activated immune cells, cytokines, and other physical stimuli [23]. Intracellular ROS consist of superoxide, hydrogen peroxide (H_2_O_2_), and hydroxyl radicals that are derived from uncoupled eNOS, NADPH oxidase, electron transport chain in mitochondria, and xanthine oxidase (Figure 1) [24,25]. Under controlled environments, these ROS serve as second messengers regulating cell proliferation, differentiation, immune responses, and tissue repair [26]. However, an overabundance of ROS leads to EC dysfunction, increasing the risk of atherosclerosis, hypertension, hypercholesterolemia, stroke, diabetes, obesity, and other metabolic syndromes [27,28]. 

### 2.1. Uncoupled eNOS

In ECs, the eNOS enzyme produces NO, an unorthodox, highly reactive messenger molecule that regulates vascular tone, gene transcription, mRNA translation, and post-translational modifications of proteins [29]. eNOS requires L-arginine and molecular oxygen (O_2_) to produce NO along with the cofactors tetrahydrobiopterin (BH4), nicotinamide adenine diphosphate (NADPH), flavin mononucleotide, heme, and flavin adenine dinucleotide [30]. Depletion of these molecules leads to eNOS uncoupling, where O_2_^−^ is produced in abundance rather than NO. Superoxide reacts with NO to generate peroxynitrite (ONOO^−^). This unstable molecule rapidly oxidizes BH4, which then maintains the state of eNOS uncoupling and reduces NO bioavailability [30,31]. This rapid oxidative inactivation of NO and overproduction of ROS by uncoupled eNOS has been shown to increase the risk of developing vascular disease factors [32,33]. Our team observed that a poorly characterized G protein-coupled receptor, GPR35, possesses an inhibitory role in eNOS activation and GCH1-mediated BH4 synthesis [34], but the exact molecular basis for the multifactorial regulation of eNOS in aortic ECs remains elusive. 

### 2.2. NADPH

NADPH oxidase is an enzyme whose primary function is to catalyze the production of superoxide from oxygen and NADPH. ROS produced from NOX isoforms are involved in proliferation, migration, and differentiation in ECs [35,36], and are known to cause mitochondrial DNA damage, induce oxidative inactivation of BH4, stimulate the conversion of xanthine dehydrogenase (XDH) to XO, and manipulate the opening of the mitochondrial ATP-sensitive K^+^ ion channel (mitoK_ATP_) [36]. The NOX family has seven isoforms, known as NOX1/2/3/4/5 and dual oxidase 1/2 (DUOX1/2). Many have been found to induce vascular dysfunction and inflammation [37]. NOX1 and NOX2 generate superoxide as opposed to NOX4, which is constitutively active and converts superoxide to H_2_O_2_ (Figure 1) [38,39]. In addition, NOX2 oversees the transfer of electrons from cytosolic NADPH to molecular oxygen and participates in signal transduction, angiogenesis, and cell death [40]. NOX3, although also responsible for generating ROS, is mainly expressed in the inner ear and has not been observed as a key factor in EC dysfunction and vascular damage [41]. NOX5 is the only calcium-regulated isoform present in the endothelium and has been found to interact with glucose and angiotensin II [42]. In low concentrations of Ca^2+^, NOX5 enhances ROS production in ECs [40]. DUOX1 and DUOX2 possess roles in immune response and cell differentiation, with both being expressed in the stomach, lungs, and thyroid. Their functions in the vascular system are relatively uncertain, although they are responsible for H_2_O_2_ generation [43]. Overall, these enzymes serve as primary sources of ROS in ECs and require further investigation in metabolic disorders and the vascular system.

### 2.3. Mitochondrial Electron Transport Chain

Mitochondria are crucial for the generation of ATP in cells. In ECs, mitochondrial-derived reactive oxygen species (mROS) are critical for cellular responses to vascular risk factors. Leakage of electrons from the mitochondrial electron transport chain generates superoxide. The production of mROS by the ETC is tightly regulated in order to avoid oxidative damage to cellular processes [44]. In the inner membrane, electron transport chain complexes I-IV generate a proton gradient that drives the production of ATP [25]. First, electrons (e^−^) from NADH and FADH2 pass through complexes I and II into complex III by ubiquinol (CoQ). Cytochrome c then transfers the electrons to complex IV, where O_2_ is reduced into H_2_O. Protons reenter the mitochondrial matrix through complex V and are used to generate ATP [45]. During this cycle, electrons are leaked from complexes I and III, and superoxide is then generated toward the matrix and intermembrane space [45,46]. The increase in superoxide results in the swelling of mitochondria and further membrane breakdown, leading to the release of cytochrome C. The latter then triggers a caspase cascade and induces cell apoptosis [47]. The mitochondria dysfunction of this organelle is one of the major causes of ROS overproduction and EC dysfunction, making it a potential target for CVD treatments.

### 2.4. Xanthine Oxidases

Xanthine oxidoreductase (XOR) is a terminal enzyme that produces superoxide and hydrogen peroxide by catalyzing the oxidation of hypoxanthine, which is then converted to uric acid (UA) [48,49]. XO and xanthine dehydrogenase (XDH) are the two interconvertible forms of XOR where NAD+ is used to generate superoxide and peroxide [50,51]. Though this enzyme is one of the major sources of ROS, it is mainly studied due to its ability to produce uric acid. UA is the end-product of purine metabolism. Under normal physiological conditions, UA possesses antioxidant activity and can aid in the regulation of ROS generated by XOR by reacting with ONOO^−^ to form NO-releasing derivatives or by targeting the Nrf2 pathway [52,53,54]. When synthesis and excretion are unbalanced, hyperuricemia can occur [52] and cause inflammation and EC dysfunction, eventually progressing into gout, atherosclerosis, or chronic kidney disease if left untreated [54]. Although an increase in XOR levels can lead to damaging ROS, the conversion of these molecules into uric acid can provide oxidative benefits.

### 2.5. Antioxidant/Defense Systems in ECs for ROS

Antioxidants counteract and eliminate ROS, making them critical molecules to defend against oxidative stress in ECs. The two types of antioxidants include enzymatic and non-enzymatic, where enzymatic antioxidants directly catalyze ROS and non-enzymatic either promote anti-oxidative enzymes or aid in oxidative chain reactions [55]. Superoxide dismutase (SOD), catalase (CAT), and glutathione peroxidase (GPx) are the main enzymes involved in ROS metabolism. SODs, which catalyze the conversion of superoxide to hydrogen peroxide, are composed of three isoforms, including copper–inc superoxide dismutase (Cu/ZnSOD, SOD1), manganese superoxide dismutase (MnSOD, SOD2), and extracellular superoxide dismutase (EcSOD, SOD3) [56]. Cu/ZnSOD is an active dimer that converts superoxide into molecular oxygen and hydrogen peroxide using copper and zinc as its metal cofactors [57]. MnSOD, the greatest source of cellular ROS of the three isoforms, regulates ROS in the mitochondrial matrix [58]. EcSOD is the least-studied isoform but has thus far been shown to exert anti-inflammatory abilities, regulate cell barrier function, modulate cytokine response, and protect tissues against ROS [59]. In the mitochondrial matrix, superoxide is converted to H_2_O_2_ by SOD2, while intermembrane space superoxide is converted by SOD1 after it passes through voltage-dependent anion channels (VDAC) [60]. Catalase is an Fe-containing enzyme that catalyzes hydrogen peroxide into H_2_O and molecular oxygen [61]. Heme-containing enzymes, true catalases, catalase-peroxides, and manganese catalases are used to classify this enzyme [62]. Yang et al. found that inhibiting catalase in ECs promotes ROS production and enhances oxidative damage under a hypoxic environment [63]. Similar to catalase, GPx is also responsible for reducing hydrogen peroxide and catalyzing GSSG, with GSH as a reductant [64,65]. Prasai et al. inhibited glutathione reductase activity and observed enhanced protein S-glutathionylation, VEGFR2 activation, and increased reactive oxygen species (ROS) production in aortic ECs, demonstrating that the cellular GSH: GSSG ratio is critical in managing oxidative stress [66]. Antioxidant enzymes can be impaired in many metabolic disorders, particularly in hyperglycemia, due to multifactorial mechanisms [67]. They have become therapeutic targets as restoration of their ability to reduce oxidative stress and prevent EC dysfunction provides a path toward reducing the risk of developing cardiometabolic disorders.

## 3. Pathways Regulated by ROS

In this section, we discuss oxidant and antioxidant pathways that are primarily regulated by intracellular ROS production [68], including two pathways that have garnered increasing interest involving transcription factors NF-κB and Nrf2 [69,70]. 

### 3.1. Regulation of NF-κB

**Figure 2:**
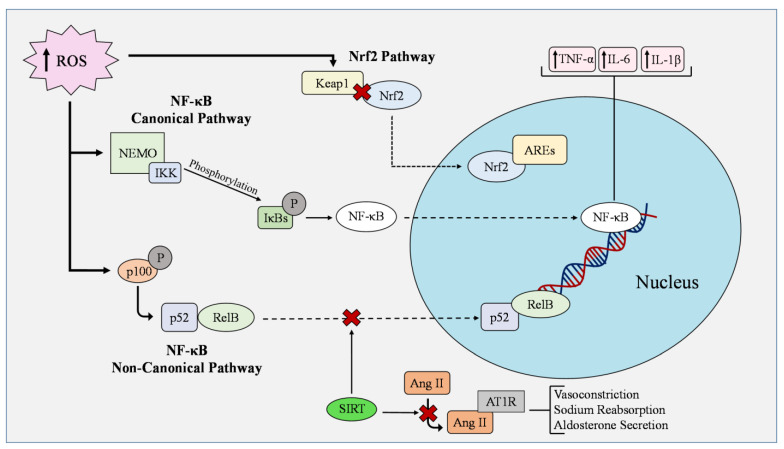
Antioxidant and oxidant pathways in endothelial cells in cardiometabolic disorders. In the nuclear factor kappa B (NF-κB) canonical pathway, IkappaBs (IκBs) are phosphorylated by the IκB kinase (IKK) complex in response to inflammatory signals activating the NEMO-binding domain. The degradation of the IκBs then allows the nuclear transport of NF-κB proteins, which initiates gene expression. In contrast, the non-canonical pathway aims to activate the NF-kB transcription factor RelB/p52 complex by phosphorylating and processing p100 into p52, where it can then bind to RelB. NF-κB regulates the expression of tumor necrosis factor α (TNF-α), interleukin-6 (IL-6), and interleukin-1β (IL-1β). The Nrf2/Keap1–ARE pathway is one of the essential antioxidant pathways for ROS management and it is activated by laminar sheer stress in ECs. Under conditions of oxidative stress, ROS interact with Kelch-like-ECH-associated protein 1 (Keap1) and hinders the ubiquitination of nuclear factor erythroid 2 related factor 2 (Nrf2). Nrf2 then translocates into the nucleus and binds to antioxidant response elements (AREs). Keap1 can turn off this signaling if ROS levels decrease. Sirtuin 1 (SIRT1) plays an inhibitory role in regulating the activity of p53 and NF-κB pathways while other family members manipulate the activation of angiotensin II type 1 receptor (AT1R). Angiotensin II (Ang II) activates AT1R through direct binding, leading to vasoconstriction, sodium reabsorption, and aldosterone secretion.

The nuclear transcription factor-κB (NF-κB) regulates inflammation, proliferation, and apoptosis in vascular ECs and also participates in gene expression, immune responses, and modulation of ROS production [71,72]. The NF-κB network consists of five protein monomers composed of homodimers and heterodimers, all of which bind differently to DNA. Two pathways regulate these proteins: the canonical (classical) pathway, which is NF-κB essential modulator (NEMO)–dependent, and the non-canonical (alternative) pathway, which is NEMO-independent [73]. In the canonical pathway, IkappaBs (IκBs) are phosphorylated by the IκB kinase (IKK) complex in response to inflammatory signals activating the NEMO-binding domain (Figure 2). The degradation of the IκBs then allows the nuclear transport of NF-κB proteins, which initiates gene expression [73,74]. In contrast, the non-canonical pathway aims to activate the NF-kB transcription factor RelB/p52 complex by phosphorylating and processing p100 into p52, where it can then bind to RelB [75]. Though NF-κB can be activated by a variety of cellular events, its most robust activators are tumor necrosis factor α (TNF-α), lipopolysaccharide (LPS), and interleukin-1 (IL-1); increasing evidence has suggested that ROS, especially H_2_O_2_, can activate the NF-κB pathway by directly modifying NF-κB heterodimers or by oxidation of its upstream kinases such as IKK [72]. While NF-κB induces the expression of many antioxidant molecules that cope with ROS, such as MnSOD, Ferritin Heavy Chain, Glutathione S-transferase pi (GSTP1), Metallothionein-3, NAD(P)H quinone dehydrogenase 1, HO-1, and GPx-1 [76], the activation of NF-κB in hyperglycemia triggers the transcriptions of proinflammatory cytokines and adhesion molecules, and also p38/JNK pathway activation, switching EC to proinflammatory and pro-coagulatory and prothrombotic phenotypes [77]. This collective evidence suggests that, although the normal physiological activity of NF-κB has the potential to reduce oxidative stress, reducing its expression in high-glucose conditions will benefit vascular diseases.

### 3.2. Regulation of Nrf2

Nuclear factor erythroid 2-related factor 2 (Nrf2) is a transcription factor that binds to antioxidant response elements (AREs), promotes the transcription of antioxidants, induces ROS-detoxifying enzymes, and regulates metabolic reprogramming for amino acids, lipids, heme, aerobic glycolysis, and glycogen synthesis [78,79]. This transcription factor has seven NRF2-ECH (Neh) domains that modulate Nrf2 activity and transcription. The Neh1 domain contains interactive proteins that recognize AREs for gene transcription, while Neh2 interacts with the Kelch-like-ECH-associated protein 1 (KEAP1) to mediate Nrf2 degradation. Neh3/4/5 primarily function as transcription activation domains, and Neh6 contains two redox-independent degrons, DSGIS and DSAPGS, that mediate NRF2 degradation by binding to E3 ubiquitin ligase β-transducin repeat-containing protein (βTrCP). Finally, the last domain, Neh7, functions in repressing NRF2 activity [79].

The Nrf2/Keap1–ARE pathway is one of the essential antioxidant pathways for ROS management and is activated by laminar sheer stress in ECs (Figure 2). Under conditions of oxidative stress, ROS interact with Keap1 and hinders the ubiquitylation of Nrf2. Nrf2 then translocates into the nucleus and binds to AREs. Keap1 can turn off this signaling if ROS levels decrease [80]. Geraniol is an antimicrobial agent in various plants’ extracted oils [81]. In oxidized low-density lipoprotein (Ox-LDL)-treated HUVEC, Geraniol reduced the expression of proinflammatory vascular adhesion molecules and lowered the production of ROS by regulating Nrf2 through PI3K/AKT signaling [82]. Increasing the expression and activity of Nrf2 and its pathways may have therapeutic potential in upkeeping the binding of AREs, thereby producing more antioxidant effects and protecting endothelial cell function.

## 4. Endothelial Cell Damage by ROS in Metabolic Disorders

Oxidative stress is tightly controlled within vascular ECs. Antioxidants typically maintain it at very low levels to balance redox signaling [83]. However, ROS at high concentrations can cause EC dysfunction, enhance inflammation and fibrosis, and induce lipid peroxidation [84,85]. Abnormal production of ROS in endothelial cells has been linked to various CVD risk factors found in metabolic disorders such as Ox-LDL, hyperglycemia, homocysteine (Hcy), hypoxia, hydrogen peroxide, and reactive aldehydes [86,87]. eNOS uncoupling and superoxide overproduction disrupts cardiac contractility and induces myocardial hypertrophy by influencing calcium ion channels [88]. By damaging ECs, ROS also influence apoptosis. Through intrinsic and extrinsic pathways, cell destruction is induced by the activation of caspases. These proteases are categorized as either initiators or effectors (executioners), where initiators cleave the inactive forms of effector caspases to begin the downstream event of apoptosis [89,90]. These changes to vascular homeostasis aggravate EC abnormalities and increase the risk of metabolic syndrome, including insulin resistance (IR), diabetes, hypertension, hyperlipidemia, obesity, and nonalcoholic fatty liver disease [91,92]. Understanding how ROS create ties between EC dysfunction and cardiometabolic disorders is critical to prevent disease progression and develop treatments for CV events. 

Biomarkers of oxidative stress in ECs that have been identified in human clinical trials include fluctuating levels of myeloperoxidase (MPO), Plasma F2-isoprostanes, biopyrrins, glutathione peroxidase 1, 8-Hydroxyl-2′-deoxyguanosine, malondialdehyde (MDA), nitrotyrosine, and antioxidant status [93,94]. In finding treatments for ROS damage, studies have turned to the investigation of these biomarkers and EC pathways, including SIRT1, NF-κB, Nrf2, AMPK, PI3K, and AKT. 

## 5. ROS in Endothelial Cells under Metabolic Stress 

**Figure 3:**
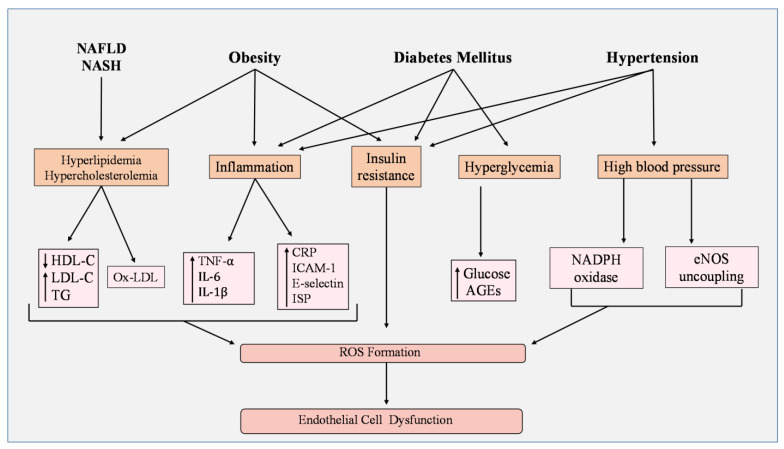
Metabolic disorders and ROS. Hyperlipidemia and hypercholesterolemia presented in non-alcoholic fatty liver disease (NAFLD) and non-alcoholic steatohepatitis (NASH) induce an imbalance in cholesterol levels by lowering high-density lipoprotein cholesterol (HDL-C) and increasing low-density lipoprotein cholesterol and triglycerides. Additionally, oxidized LDL (Ox-LDL) is also enhanced. Obesity not only influences cholesterol levels but also creates inflammation and insulin resistance. Inflammation has been shown to increase the activity of oxidant biomarkers including C-reactive protein (CRP), intercellular adhesion molecule-1 (ICAM-1), E-selectin, and F2-isoprostanes (ISP). Proinflammatory cytokine secretion is enhanced, such as tumor necrosis factor-α (TNF-α), interleukin 1β (IL-1β), and interleukin-6 (IL-6). In diabetes mellitus (DM), hyperglycemia develops along with inflammation and insulin resistance, leading to high levels of glucose and advanced glycation end-products (AGEs). Finally, hypertension, through high blood pressure, can activate NADPH oxidase and induce endothelial nitric oxide synthase (eNOS) uncoupling, while impairing insulin signaling and causing inflammation. Collectively, these factors stimulate the overproduction of reactive oxygen species (ROS) and cause endothelial cell (EC) dysfunction.

ROS overproduction and EC dysfunction are recognized as biomarkers in metabolic disorders and contribute to disease progression [95,96]. Current studies have investigated the impact of manipulating antioxidant expression and oxidant pathways in metabolic disorders, including hypertension [97], obesity [98], diabetes mellitus [99], hyperlipidemia [100], and nonalcoholic fatty liver disease [101]. Understanding how EC damage and oxidative stress induce cardiometabolic events may reveal new ways to better current treatments or create new and effective therapies (Figure 3).

### 5.1. Hypertension 

Hypertension is characterized by high blood pressure and contributes to mortality incidences worldwide [102]. ROS overproduction and EC dysfunction induce various CV changes in hypertension, including vascular remodeling, increased vasoconstriction and lipoprotein oxidation, thrombus formation, post-translational modification, and downstream activation of target proteins [18,102]. Multiple redox pathways have emerged as key targets in managing oxidative stress in hypertension studies. SIRT1 has been shown to directly interact with eNOS through its deacetylation and activation of eNOS [103] or by increasing eNOS transcription in forkhead box O (FOXO)1-dependent or Krüpple link factor 2 (KLF2)-dependent manners [104,105,106]. Additionally, the activation of NOX isoforms leads to decreased SIRT1 activity and NO production, resulting in increased ROS. Furthermore, recent studies indicate that SIRT1 plays an inhibitory role in regulating the activity of p53 and NF-κB pathways, while other SIRT family members, such as SIRT2 and SIRT3, interact with angiotensin II (Ang II) type 1 receptor (AT1R), FOXO1, and SOD signaling [107,108,109,110]. Angiotensin II activates AT1R through direct binding, leading to vasoconstriction, sodium reabsorption, and aldosterone secretion [111]. The excessive activation of AT1R enhances these effects and induces hypertension [112]. Therefore, manipulation of SIRT may aid in the front lines of anti-hypertensive drugs Ang II-converting enzyme inhibitors or Ang II receptor blockers. 

### 5.2. Obesity

Abdominal fat accumulation contributes to pro-oxidant and inflammatory states, making obesity one of the major risk factors for diabetes mellitus (DM), hyperlipidemia, and non-alcoholic fatty liver disease (NAFLD) [113]. Adipose tissue, which expands and undergoes remodeling during obesity, contributes to vascular EC dysfunction by secreting vasoconstrictor mediators and proinflammatory cytokines such as TNF-α, interferon-gamma (IFNγ), interleukin-1β (IL-1β), and interleukin-6 (IL-6) [113,114,115,116]. In a study conducted on metabolic syndrome, groups with obesity and insulin resistance showed significantly lower levels of SOD enzyme activity [117]. Oxidative stress has been shown to induce many of these risk factors [118]. Many features that are used to screen for prediabetes, such as insulin resistance, impaired beta-cell function, elevated blood pressure and triglyceride levels, low HDL cholesterol, free fatty-acid (FFA) accumulation, and unbalanced adipokine and cytokine secretion [119], are associated with endothelial cell dysfunction [120,121,122]. Regulating oxidative stress may become a possible method to prevent the progression of obesity-related effects into metabolic disorders. 

### 5.3. Diabetes Mellitus 

Diabetes mellitus is a chronic hyperglycemic condition that occurs either when the pancreas does not produce enough insulin or when the body cannot effectively use insulin to regulate glucose [123,124]. EC dysfunction is a common factor found in diabetic patients as EC health can be damaged by various CV risk factors such as insulin resistance, excessive free fatty-acid accumulation, protein kinase C activation, overexpression of growth factors and cytokines, and macrophage polarization [125,126,127]. In direct relation to EC dysfunction and oxidative stress, FFA accumulation impairs eNOS phosphorylation and generates ROS, thereby activating the NF-κB pathway and increasing inflammation [128]. In normal physiological cell processes, fatty acids are oxidized and act as a source of ATP. However, under diabetic conditions, FA is stored, which leads to reduced ATP production and cardiac contraction [129]. Huang et al. found that high fatty-acid metabolism and β-adrenoceptor activation promotes myocardial ROS production, cardiac dysfunction, and calcium overload in hypoglycemic mice [130]. Additionally, new markers for oxidative stress and EC dysfunction were associated with type II diabetes (T2D), including F2-isoprostances, intercellular adhesion molecule-1 (ICAM-1), and E-selectin [131]. Furthermore, reduced antioxidant status and enhanced proinflammatory markers (TNF-α, IL-6, IL-1β) were observed in diabetic rats [132].

Studies have shown that manipulating ROS pathways could be a plausible treatment for DM. Han et al. observed that acacetin, an O-methylated flavone, alleviated oxidative stress, reduced cell apoptosis, and attenuated mitochondrial injury in ECs by activating SIRT1/SIRT3/AMPK signals [133]. AMP-activated protein kinase (AMPK) is an energy-sensing enzyme that responds to nutrient depletion and abnormal ATP levels by increasing fatty-acid oxidation and glucose transport (Figure 3) [134,135]. Hu et al. found that salidroside, an active compound within Chinese herbal medicine, decreased levels of inflammatory cytokines, promoted antioxidant activity, and inhibited the generation of ROS in advanced glycation end-product (AGE)-induced human umbilical vein ECs (HUVECs) via the AMPK/NF-κB/NLRP3 signaling pathway [99]. Evidence suggesting that AMPK and NLRP3 are good targets for the NF-κB pathway is growing. Stress adaptation for ECs is increased upon AMPK activation, which has been shown to require a mechanistic target of rapamycin complex 1 (mTORC1) coupling by eNOS [136]. On the other hand, the upregulation of NLRP3 inflammasomes leads to pyroptosis in ECs [137]. While increasing AMPK activation would be beneficial for EC health, inhibiting inflammation induced by NLRP3 would also influence the NF-κB pathway and reduce oxidative stress. 

### 5.4. Hyperlipidemia

Lipids and lipoproteins are required for normal physiological functions. For example, lipid membrane rafts are pivotal structures that consist of specific lipid components and proteins that host a variety of membrane-associated enzymatic complexes, including NADPH oxidase, the activation of which produces ROS. Having abnormal levels of low-density lipoprotein cholesterol (LDL-C), high-density lipoprotein cholesterol (HDL-C), and triglycerides (TG) in the blood due to imbalanced fat metabolism is known as hyperlipidemia [138,139]. Hypercholesterolemia (elevated LDL-C) is a pro-atherosclerotic disease [140] associated with oxidative stress in ECs. In hyperglycemia, extracellular free cholesterol can be directly incorporated into the plasma membrane, increasing cellular cholesterol levels. The increase in plasma membrane-free cholesterol leads to modified reactions that enhance NADPH oxidase-derived ROS production [141]. On the other hand, excessive ROS induce LDL oxidation [142]. The oxidized LDL (Ox-LDL) can increase its macrophage uptake by binding to scavenger proteins [143]. This process damages the vascular endothelium by causing inflammation and apoptosis [144]. In two different clinical studies on familial hypercholesterolemia (FH), patients possessed higher markers for oxidative stress, such as MDA and MPO, than healthy groups [145,146]. Overall, these studies propose that the excess LDL accumulation and Ox-LDL formation may contribute to oxidative stress by increasing the activity of oxidant biomarkers, including high-sensitivity C-reactive protein (hsCRP), soluble intercellular adhesion molecule-1 (sICAM-1), E-selectin, and F2-isoprostanes (ISP) [145]. Considering this, it is imperative that Ox-LDL be reduced in patients with hyperlipidemia to prevent ROS-induced EC dysfunction. 

### 5.5. Non-Alcoholic Fatty Liver Disease 

Non-alcoholic fatty liver disease is the accumulation of fat in the liver that is not caused by alcohol consumption but by other inducers of liver failure [147]. NAFLD is diagnosed based on non-alcoholic fatty liver (NAFL) severity and non-alcoholic steatohepatitis (NASH). NAFL is the buildup of fat in the liver without hepatocellular injury, while NASH is the presence of hepatic steatosis combined with inflammation and liver injury [148]. This disease represents a chronic risk factor closely linked to metabolic disorders. According to recent observations, NASH progresses markers for oxidative stress and endothelial dysfunction in patients with NAFLD by elevating cytokine secretion and inflammation (Figure 3) [92,149]. Li et al. found that hesperetin, a flavonoid found in citrus fruits, alleviates oxidative stress under in vivo hepatotoxic conditions by increasing Nrf2 activation through the PI3K/AKT–Nrf2–ARE pathway [150]. Others have discovered that resveratrol (3,5,4′-trihydroxy-trans-stilbene), a natural phenol compound with antioxidant characteristics, normalizes NO production levels by reducing NADPH oxidase expression and improving SIRT1 activity [151,152]. Targeting oxidative pathways has shown promise in treating NAFLD risk factors by reducing ROS production and NO bioavailability.

## 6. Redox-Modulation of Current Market Drugs 

Current market drugs for cardiometabolic disorders have shown success in treating symptoms. However, drug-induced oxidative stress has caused concern about whether the adverse effects created by a drug outweigh its benefits. Reactive intermediates produced during drug metabolism can reduce molecular oxygen into ROS, leading to toxicity in multiple organ systems [153,154]. Morphine and Ibuprofen are widely taken for pain management but have been shown to increase ROS production in ECs with increased exposure, indicating that these drugs may not cause toxicity except for long-term use [155,156]. Specifically, morphine has demonstrated a role in increasing the activity of caspase-3, caspase-9, IL-1β, TNF-α, and IL-6 [157]. Atorvastatin, a commonly used drug for high cholesterol, has demonstrated various effects on redox status in cardiomyocytes by either inducing or alleviating ferroptosis [158,159]. However, in endothelial cells, Dang et al. found that ROS induced by Ang II were reversed with atorvastatin treatment [160]. Whether statins maintain or harm redox status requires further investigation, as individual cell types may have different interactions. While some drugs induce oxidative stress, others have been shown to reduce it. Studies have consistently demonstrated that resveratrol, a natural polyphenol, protects against oxidative stress by interacting with NF-κB, SIRT1, inflammatory cytokines, and various oxidative microRNAs [161,162]. Furthermore, prolonged exposure to resveratrol in alcohol-dependent mice demonstrated an antioxidant effect that prevented ROS formation [163]. Additionally, inducing GLO1 expression with trans-resveratrol and hesperetin improved wound closure and angiogenesis in vivo [164]. Understanding which drugs impact ROS generation and antioxidant expression is critical for future treatments, as dosage and exposure time can be adjusted to better protect patients from adverse reactions.

## 7. MicroRNAs and ROS 

**Figure 4:**
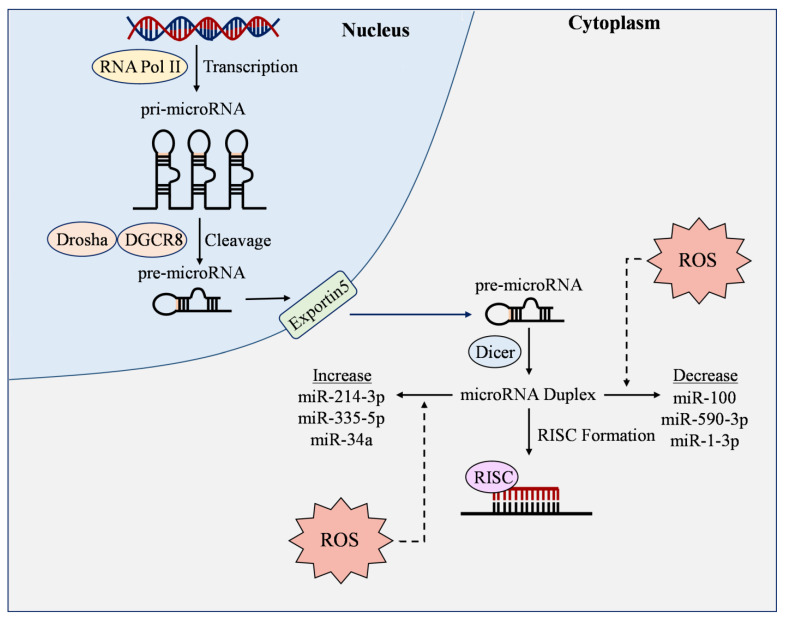
ROS-responsive microRNAs. RNA Polymerase II (RNA Pol II) transcribes genes into pri-microRNA (pri-miRNA), where they are then cleaved by Drosha and DGCR8 into pre-microRNA (pre-miRNA). Pre-microRNA is then transported out of the nucleus by Exportin5, where it is cleaved by Dicer to produce mature microRNA duplex. An RNA-induced silencing complex (RISC) then forms to bind with the 3′-untranslated region of messenger RNAs to exert miRNA functions. During this process, reactive oxygen species (ROS) can increase or decrease the expression of microRNAs, thereby manipulating their ability to regulate oxidative stress and other molecules in endothelial cells.

MicroRNAs are small non-coding RNA at ~20 nt in length that targets the 3′untranslated region of messenger RNAs and regulate gene expression at post-transcriptional levels. MiRNAs can have either positive or negative effects on ROS production by controlling genes regulating ROS biogenesis and scavenging [165]. On the other hand, ROS co-regulate with miRNAs by modulating their transcription and maturation (Figure 4) [166]. Growing evidence suggests that studying the functional relationship between redox-regulating enzymes, antioxidant pathways, and miRNAs may be critical to developing treatments for metabolic and cardiovascular illnesses [167].

### 7.1. ROS-Responsive MicroRNAs 

ROS-responsive miRNAs have demonstrated varying expression levels under oxidative stress. Gu et al. found that the upregulation of leucine-rich containing family pyrin domain containing 1 (NLRP1)/NOX4 led to decreased levels of miR-590-3p in human retinal microvascular ECs [168]. By inhibiting miR-590-3p, they discovered that NLRP1, caspase-1, and intracellular ROS were increased under hyperglycemic conditions [168]. Other studies have observed the connection between ROS and miRNAs by directly increasing H_2_O_2_. Wang et al. found that miR-100 expression was decreased in H_2_O_2_-treated HUVECs and that overexpressing this miRNA protected ECs against inflammation and oxidative stress by inactivating Notch signaling [169]. In another report, HUVECs that were exposed to H_2_O_2_^−^, high glucose, or TNF-α showed upregulated expression of miR-335-5p [170]. SIRT7, which has previously been shown to have a role in reducing ROS production [171], was markedly decreased in H_2_O_2_-induced ECs, and premature senescence was observed [170]. Inhibition of miR-335-5p improved SIRT7 expression, which then alleviated EC dysfunction caused by oxidative stress [170]. In a similar model, the excessive advanced glycation end-products (AGEs) reduced miR-1-3p which targeted and repressed myosin light chain kinase in HUVECs, leading to impaired EC layer integrity [172]. 

In human aortic ECs transfected with Ox-LDL, oxidative stress biomarkers are exacerbated [173]. However, miR-486-5p overexpression reversed these factors and suppressed apoptosis [173]. Similar results were observed with overexpressed miR-20a, which reduced ROS generation after ECs had been exposed to Ox-LDL [174]. Other microRNAs have shown opposite effects in DM studies. MiR-34a was upregulated by Ox-LDL treatment in HUVECs, and knockdown of this miRNA improved mitochondrial membrane potential, diminished caspase-3 activity, and suppressed intracellular ROS [175]. Xie et al. observed a similar detrimental effect as Ox-LDL treatment increased miR-214-3p expression and lowered GPx4 activity [176]. Inhibition of miR-214-3p restores cell migration and reduces ROS levels [176]. Observation of ROS-responsive miRNAs has demonstrated a clear connection between miRNA expression, high ROS levels, and EC protection, indicating that these molecules could be potential targets in preventing metabolic disorders.

Current trials that are recruiting, or have not published their completed data, have examined oxidative stress in cardiometabolic disorders by manipulating microRNAs, including miR-210 (NCT04089943), miR-146a (NCT04583085), miR-126 (NCT01875484), miR-133a (NCT05692752), miR-200b (NCT05383794), miR-21 (NCT02581098), miR-155 (NCT04277390), and miR-208b (NCT05692752). With success, these molecules may become important targets for future treatments.

### 7.2. Regulation of ROS by MicroRNAs

Studies have found that ROS and miRNAs co-regulate each other, indicating a relationship that could be exploited for therapeutic purposes. Antioxidant pathways that have garnered increased interest involve Nrf2, ARE, and members of the SIRT family. Inhibition of miR-34a and miR-383 can suppress oxidative stress and improve EC function under hyperglycemic conditions by increasing SIRT1 expression [177,178]. Hu et al. found that miR-383 suppression elevated CAT and SOD1 activity in HUVECs and the aortas of diabetic mice [178]. Overexpression of miR-126 suppresses ROS and MDA levels and increases SOD and GPx activity in HUVECs by promoting SIRT1/Nrf2 signaling [179]. In Ox-LDL-treated HUVECs, overexpression of miR-140-5p increased ROS and MDA production, while antioxidant activity for SOD, GSH, and GSH-Px was reduced [180]. When exposed to Ang II, miR-4432 targets fibroblast growth factor binding protein 1 in human brain microvascular ECs to reduce mitochondrial ROS production [181]. Some miRs have been found to protect ECs from excessive oxidative stress under hyperglycemic conditions. For example, expression and transcription activity of the Nrf2/ARE pathway was downregulated when cells were transfected with a miR-602 inhibitor [182]. In contrast, oxidative damage observed in ECs with the inhibitor was prevented with high expression of miR-602 [182]. MiR-361-5p has been reported to target tumor necrosis factor receptor-associated factor 3 to protect mouse retina microvascular ECs against oxidative stress in a high-glucose environment [183]. In diabetic rats, miR-24 demonstrated decreased expression during vascular injury and was accompanied by reduced levels of Nrf2 and *heme oxygenase*-*1* (HO-1) [184]. ROS production, MDA, and NOX activity were reduced after miR-24 overexpression [184]. 

**Figure 5:**
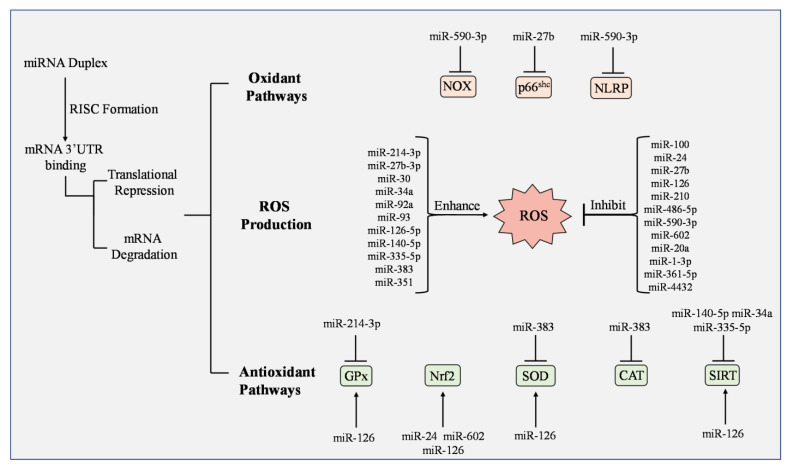
Regulation of ROS production, oxidant pathways, and antioxidants by microRNAs. When mature microRNAs (miRNA) form an RNA-induced silencing complex (RISC), translational repression or mRNA degradation can occur and either enhance or inhibit ROS production. NADPH oxidase (NOX), p66shc, and NLRP participate in oxidant pathways and are inhibited or reduced by miR-590-3p and miR-27b, respectively. Studies have observed greater impact on antioxidants by miRNAs. Glutathione peroxidase (GPx), nuclear factor erythroid 2-related factor 2 (Nrf2), superoxide dismutase (SOD), and various forms of Sirtuin (SIRT) have increased activation by multiple miRNAs including miR-125, miR-24, and miR-602. Some miRNAs have demonstrated inhibitory effects on antioxidants including miR-214-3p, miR-383, miR-140-5p, miR-34a, and miR-335-5p.

In addition to modulating molecules in redox pathways, miRNAs have other mechanisms to regulate ROS production (Figure 5). 25-hydroxyvitamin D_3_ (25(OH) D_3_) is a form of vitamin D that downregulates intracellular ROS-related pathways when concentrations are at normal levels [185]. In retinal microvascular ECs, overexpression of miR-93 inhibited 25 (OH) D_3_ activity. This led to increased ROS production and Fe^2+^ levels [186]. In vivo models with microRNAs have also shown success. Inhibition of miR-27b-3p decreased oxidative stress in hypoxic ECs and alleviated myocardial ischemia/reperfusion injury in rats [187]. On the other hand, Wang et al. found that miR-27b suppresses mitochondrial ROS and p66^shc^ expression, accelerates wound closure, and improves angiogenesis in diabetic mice [188]. In a T2D mouse model where hyperglycemic lean and obese mice were evaluated, coronary microvascular ECs possessed higher levels of miR-30 compared to controls and overexpressing this microRNA increased fatty-acid β-oxidation, ROS production, and lipid peroxidation, as well as downregulated eNOS activity [189].

Since miRNAs have demonstrated a connection with regulating ROS and oxidant pathways, understanding how miRNAs prevent or develop cardiovascular and metabolic disorders is critical (Table 1). In T2D patients, miR-210 expression was lower in carotid plaques compared to the healthy control group [190]. Zhou et al. linked this to an increase in ROS production and EC dysfunction [190]. Rescuing miR-210 in T2D mice prevented EC dysfunction, identifying miR-210 as a potential therapeutic target for DM-related cardiomyopathy [190]. Additional miRNAs influencing ROS in diabetic mouse models include miR-92a and miR-351 [191,192]. Inhibition of miR-92a not only reduced ROS in diabetic mouse aortas but also increased HO-1 expression in HUVECS while silencing miR-351 alleviated atherosclerosis [191,192]. Recently, Tang et al. found that miR-92a expression was higher in streptozotocin (STZ)-induced diabetic rats and aortic ECs [193]. Administering miR-92a leads to EC dysfunction and oxidative stress by targeting the *Prkaa2* gene. Furthermore, lower levels of miR-351 expression reduced apoptosis, ROS generation, and lipid accumulation in ECs treated with Ox-LDL and high glucose [192].

While some microRNAs have consistent outcomes across multiple studies, others have contradicting results. In hypoxia-induced ECs, miR-126 mimic and VEGF-plasmid co-transfection improved EC function and AKT phosphorylation, suggesting that miRNA possesses a protective effect against EC dysfunction [194]. However, Liao et al. found that miR-126-5p knockdown inhibited oxidative stress and apoptosis [195]. Further investigation of this miRNA is therefore required as its true impact on EC dysfunction is still unknown.

## 8. Future Perspectives

Direct targeting of ROS needs to be established in specific tissues and cells. ECs play a role in physiological processes but also participate in immune response [196]. If the gene expression for a particular target molecule is primarily active in ECs, researchers must confirm that a non-specific treatment will not interact with other cell types. Manipulating specific enzymes and proteins located in ECs could provide the information needed to prevent adverse reactions between systems. Targeting microRNAs can be performed using several methods, including manipulating synthetic oligonucleotides or using different delivery systems such as virus-mediated, lipid, or polymer-based nanoparticles, extracellular vesicles (EV), inorganic carriers, and peptides [197,198,199]. Clinical studies on miRNA treatment have been conducted for cardiovascular therapy; however, such studies have struggled to succeed, as many miRNAs are not fully characterized and have many unknowns [200]. Therefore, combining different techniques may have therapeutic potential. The dual manipulation of miRNAs and oxidant/antioxidant molecular function to promote Nrf2 or inhibit NF-κB may contribute to reduced oxidative stress in multiple organ systems.

## 9. Conclusions

In this literature review, we demonstrate the role of oxidative stress in endothelial cells and metabolic disorders. Previous studies support the connection between ROS overproduction and EC dysfunction, showing that the combination of these biomarkers is a common indicator of cardiovascular risk factors and disease progression. Targeting oxidant pathways and antioxidant activity is the most promising concept for future treatments and may be attainable using microRNAs. However, though in vivo and in vitro studies suggest miRNAs play a key role in redox status, there is a lack of success in clinical trials that prove these molecules can work on their own. Therefore, it is necessary that studies investigate a combined treatment of antioxidant enhancement, or enzymatic agonist delivery and/or miRNA manipulation, to reduce oxidant pathway activity. 

## Figures and Tables

**Table 1:** MicroRNAs and oxidative stress in cardiovascular/metabolic disorders.

MicroRNA	Potential Impact on ROS	Cardiovascular/ Metabolic Disorder	Key Experimental Findings	References
miR-1-3p	reduces	Advanced glycation end-products (AGEs)	Reduced expression by AGEs impaired EC layer integrity; miR targets and represses myosin light chain kinase	[172]
miR-20a	reduces	Atherosclerosis	Ox-LDL reduces miR-20a expression in ECs; overexpressed miR-20a reduced ROS generation under Ox-LDL treatment	[174]
miR-24	reduces	Diabetes Mellitus	Activated the Nrf2/HO-1 signaling pathway	[184]
miR-27b	reduces	Diabetes	miR-27b suppresses mitochondrial ROS and p66^shc^ expression, accelerates wound closure, and improves angiogenesis	[188]
miR-27b-3p	enhances	Hypoxia	Inhibition reduced oxidative stress	[187]
miR-30	enhances	Diabetes Mellitus	Overexpression increased fatty-acid β-oxidation, ROS generation, and lipid peroxidation; expression of eNOS was also downregulated	[189]
miR-34a	enhances	Diabetes Mellitus	Inhibition reduced oxidative stress; inhibition of P53 increased miR-34a expression and reduced SIRT1 expression	[177]
miR-34a	enhances	Atherosclerosis	Knockdown protected against Ox-LDL apoptosis and ROS by inhibiting the mitochondrial apoptotic pathway	[175]
miR-92a	enhances	Diabetes Mellitus	Inhibition reduced ROS generation	[191]
miR-92a	enhances	Diabetes Mellitus Erectile Dysfunction	Inhibition reduced oxidative stress and improved EC dysfunction	[193]
miR-93	enhances	Diabetic Retinopathy	Overexpression inhibited 25 (OH) D_3_ functions by increasing ROS and upregulating Fe^2+^ levels	[186]
miR-100	reduces	Nonatherosclerotic inflammatory disease	miR-100 was decreased in H_2_O_2_-induced ECs; overexpression attenuated inflammatory response, oxidative stress, and cell apoptosis by inactivating Notch signaling	[169]
miR-126	reduces	Hypoxia	miR-126 mimic and VEGF-plasmid co-transfection improved proliferation, migration, tube-forming ability, and restored AKT phosphorylation	[194]
miR-126	reduces	Ischemia	Overexpression promoted the SIRT1/Nrf2 signaling pathway, attenuated cytotoxicity and apoptosis, decreased ROS generation and malondialdehyde content, and increased superoxide dismutase and glutathione peroxidase activity	[179]
miR-126-5p	enhances	Hypoxia	Knockdown suppressed hypoxia-induced cell apoptosis and oxidative stress in ECs	[195]
miR-140-5p	enhances	Atherosclerosis	Overexpression led to increased ROS; miR-140-5p regulated Nrf2 and SIRT2 expression	[180]
miR-210	reduces	Diabetes Mellitus Type 2	Downregulated miR-210 caused EC dysfunction	[190]
miR-214-3p	enhances	Atherosclerosis	Ox-LDL increased miR-214-3p expression and decreased GPX4 expression; overexpression of miR decreased GPX4 expression; inhibition of miR reduced ROS levels	[176]
miR-335-5p	enhances	Atherosclerosis	Overexpression decreased the SIRT7 expression in ECs and induced ROS overproduction	[170]
miR-351	enhances	Atherosclerosis	Lower levels of miR-351 expression reduced apoptosis, ROS generation, and lipid accumulation in ECs treated with Ox-LDL and high glucose	[192]
miR-361-5p	reduces	Diabetes Mellitus Obesity	Targets tumor necrosis factor receptor-associated factor 3 under high-glucose conditions	[183]
miR-383	enhances	Diabetes Mellitus	Suppression reduced ROS generation and elevated CAT and SOD1 activity	[178]
miR-4432	reduces	Hypertension	Targets fibroblast growth factor binding protein 2	[181]
miR-486-5p	reduces	Carotid artery stenosis (CAS)	Overexpression promoted EC proliferation, suppressed cell apoptosis, and reversed the release of ROS and inflammatory factors induced by Ox-LDL	[173]
miR-590-3p	reduces	Diabetic Retinopathy	Inhibition upregulated NLRP1, the NOX4/ROS/TXNIP/NLRP3 pathway, and caspase-1. NLRP1 and NOX4 were confirmed as direct target genes of miR-590-3p	[168]
miR-602	reduces	Diabetes Mellitus Type 2	Reduced ROS levels, increased Nrf2 expression, and enhanced transcription activity of Nrf2/ARE	[182]

Abbreviations: AGE, advanced glycation end-products; EC, endothelial cell; ROS, reactive oxygen species; Ox-LDL, oxidized low-density lipoprotein; Nrf2, nuclear factor erythroid 2-related factor 2; eNOS, endothelial nitric oxide synthase; SIRT, Sirtuin; GPX4, glutathione peroxidase 4; CAT, cat-alase; SOD1, superoxide dismutase; NOX, NADPH oxidase.

## Data Availability

No new data were created or analyzed in this study. Data sharing is not applicable to this article.
